# Etymologia: O’nyong-nyong virus

**DOI:** 10.3201/eid1208.ET1208

**Published:** 2006-08

**Keywords:** Etymology, Etymologia, O’nyong-nyong virus, anopheline mosquitoes

## [o-nyong′nyong]

*O’nyong-nyong* means “severe joint pain” in the language of the Acholi people of East Africa. O’nyong-nyong virus was first isolated in Uganda in 1959 at the beginning of an outbreak that spread to Kenya, Tanzania, Zaire, Malawi, and Mozambique. One of the largest arbovirus epidemics ever recorded, the outbreak lasted until 1962 and affected >2 million persons. A species of the genus *Alphavirus* and closely related to chikungunya virus, O’nyong-nyong virus is transmitted by the bite of anopheline mosquitoes and causes an acute, self-limited, febrile illness characterized by lymphadenitis and joint pain.

Sources: Dorland’s illustrated medical dictionary. 30th ed. Philadelphia: Saunders; 2003 and wikipedia.org

